# Supplementary material for: Causal interplay between lactose intolerance and gut microbiota: a combined bidirectional Mendelian randomization and in vivo validation study
Source: Front Nutr. 2026 Jun 1;13:1803337. doi: 10.3389/fnut.2026.1803337 (PMC13265576; doi:10.3389/fnut.2026.1803337)
Supplement: Supplementary file 1 [file Data_Sheet_1.zip › supplementary materials/Forward/leave one out cross validation/ebi-a-GCST90027857.finngen_R12_E4_LACTONAS.pdf]

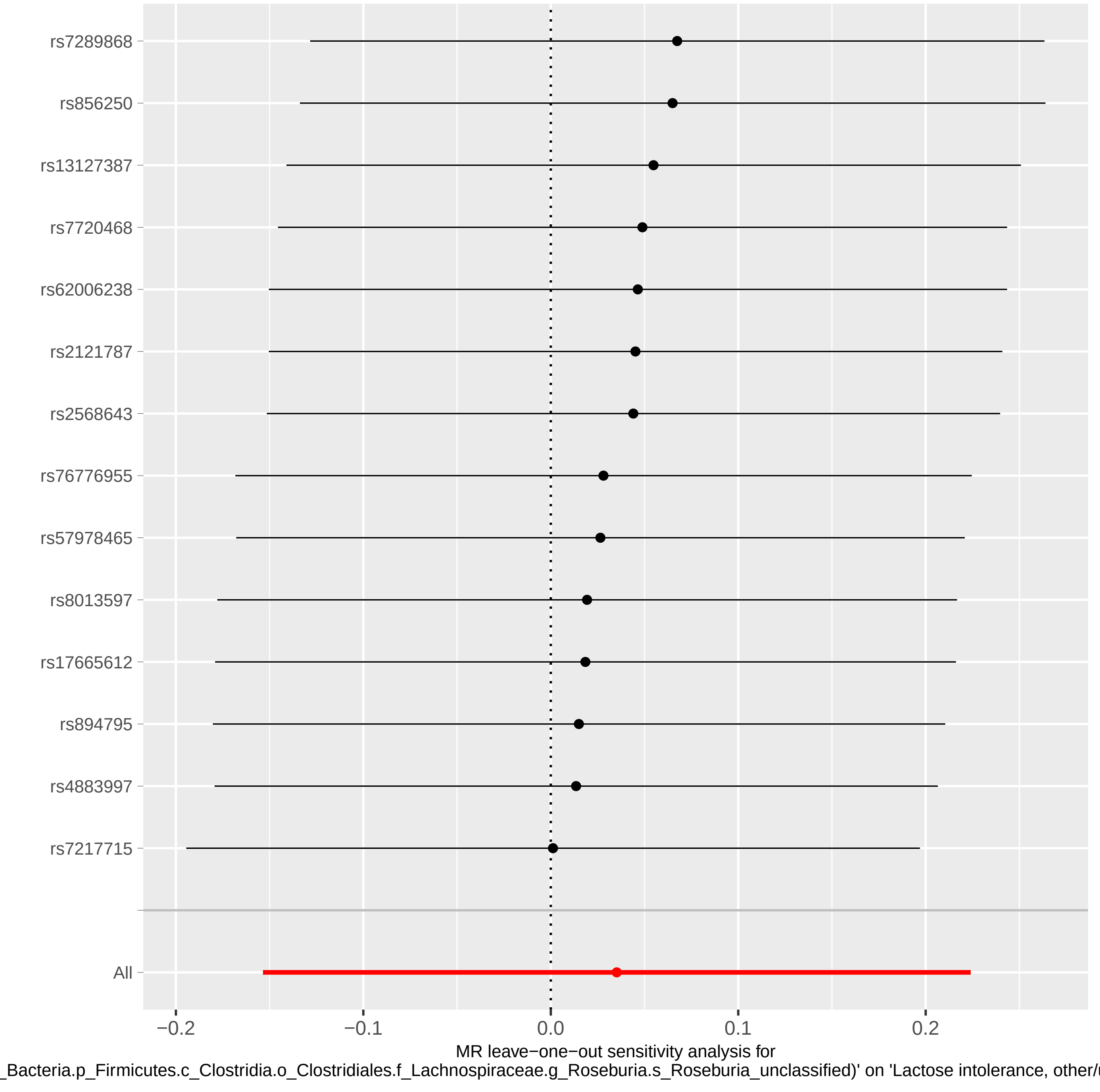

rs7289868
rs856250
rs13127387
rs7720468
rs62006238
rs2121787
rs2568643
rs76776955
rs57978465
rs8013597
rs17665612
rs894795
rs4883997
rs7217715
All
−0.2
−0.1
0.0
0.1
0.2
MR leave−one−out sensitivity analysis for
_Bacteria.p_Firmicutes.c_Clostridia.o_Clostridiales.f_Lachnospiraceae.g_Roseburia.s_Roseburia_unclassified)' on 'Lactose intolerance, other/u
